# Morphogenic Effect of Exogenous Glucocorticoid Hormones in the *Girardia tigrina* Planarian (*Turbellaria*, *Tricladida*)

**DOI:** 10.3390/biology12020292

**Published:** 2023-02-11

**Authors:** Artem Ermakov, Natalia Kudykina, Arina Bykova, Ulyana Tkacheva

**Affiliations:** 1 Institute of Theoretical and Experimental Biophysics of the Russian Academy of Sciences, 142290 Pushchino, Russia; 2 Institute of Medicine and Living System, Immanuel Kant Baltic Federal University, 236041 Kaliningrad, Russia

**Keywords:** planarian regeneration, glucocorticoid hormones, hydrocortisone, methylprednisolone, neoblast, mitotic activity

## Abstract

**Simple Summary:**

We have studied the effect of two glucocorticoid hormones: hydrocortisone and its synthetic analogue methylprednisolone on regeneration activity of *Girardia tigrina* planarian tissues. The results indicate that both hormones influence the recovery rate of the regenerating tissue in either a stimulatory or inhibitory way depending on the hormone, its concentration and the regenerating body part of the worm. We suggest that exogenous glucocorticoids can influence endogenous mechanisms of endocrine regeneration, including hormone-receptor interaction. The revealed features of the effects of hydrocortisone and methylprednisolone may be associated with the formation of specific hormone–receptor complexes with each of them, as well as with the dependence of the effects of methylprednisolone on the type of interaction with hydrocortisone receptors i.e., as an agonist or as an antagonist.

**Abstract:**

We have studied the effect of two glucocorticoid hormones: hydrocortisone and its synthetic analogue methylprednisolone on the regeneration activity of head and tail blastema of the *Girardia tigrina* planarian. The regeneration activity was studied in head and tail blastema formed after resection by means of lifetime computer morphometry and immunohistochemical labeling of neoblasts. The search for orthologous proteins—glucocorticoid receptors (hydrocortisone) was performed using the SmedGD database of the *Schmidtea mediterranea* planarian. The results indicate that both hormones influence the recovery rate of the regenerating head and tail blastema. The worms with regenerating tail blastema have less sensitivity to the hormones’ treatment compared to the ones with regenerating head blastema. Hydrocortisone at a high concentration (10^−3^ M) suppressed the regeneration rate, while stimulating it at lower concentrations (10^−4^–10^−6^ M). The same concentrations of methylprednisolone inhibited the regeneration of head blastema, but did not affect the tail blastema regeneration. The two hormones acted differently: while hydrocortisone stimulated the proliferation of neoblasts in the periwound region, methylprednisolone reduced the mitotic activity, mainly on the tail zone furthest from the wound surface. We suggest that exogenous glucocorticoids can influence endogenous mechanisms of hormone-dependent regeneration.

## 1. Introduction

Regeneration is the ability of living organisms to restore missing structures over time after damage and injury. In the last decade, planarians have been considered a promising model for regenerative medicine and stem cell biology in particular due to an extremely high content (up to 30%) of pluripotent neoblast stem cells in their body [1]. In addition, despite the relatively simple morphology, planarians have a highly structured central nervous system (CNS) and a brain complex enough to consist of all types of nerve cells, including neurosecretory [2]. Moreover, planarians have more common genes with vertebrates compared to other popular model organisms, such as *Drosophila melanogaster* or *Caenorhabditis elegans* [3]. It has been established that the conserved part of the human stem cell genome responsible for proliferation and differentiation is highly homologous (up to 90%) to the corresponding planarian genes [4]. All this makes planarians a reliable and popular model in the fields of development, regeneration and stem cell biology.

The research of humoral regulation mechanisms in the study of regeneration has a great importance in this field. In flatworms, the neurosecretory part of the endocrine system has been studied in the most detail. In their nervous system the neurons are known to be responsible for synthesis of the main mediators—serotonin (5-HT), FMRF-like peptides (GNFFRF, GYIRF, RYIRF, YIRF) and NPF-like peptides, as well as a family of pancreatic polypeptides (PP, peptide YY, substance P, neuropeptide Y) which are localized both in the central nervous system (brain ganglia and nerve trunks) and in the gonadal plexus [5,6,7,8]. There is also indirect evidence of the presence of the hormonal compounds vasopressin and oxytocin in *Microstomus lineare* flatworms [9].

The possibility of the participation of neuropeptides in the regulation of the regeneration process in different types of planaria is confirmed by the influence of certain vertebrate neuropeptides on regeneration, such as dalargin, somatostatin and vasopressin. Additionally, NPF and FMRF-like peptides can accelerate the recovery of the regenerating pharynx function, and FMRF can in certain cases, slow down this process [2]. LHRH_1-2_ and morphogen of hydra stimulate planarian regeneration. AKTH_4-10_ showed weak stimulating effect regeneration. LHRH_9-10_ and dalargin acts as a powerful inhibitor of planarian regeneration while delta sleep peptide did not have any effect on planarian regeneration [10].

In addition, representatives of free-living and parasitic flatworms are supposed to have another level of endocrine regulation—steroid hormones [11]. In different representatives of this group, some steroids, sex steroids, gluco- and mineralocorticoids, have been isolated and qualitatively and/or quantitatively determined [12,13,14].

The physiological significance of these hormones has not been sufficiently studied; however, a number of data indicate the possibility of their inclusion in the regeneration regulation process. It was shown that certain reproductive and glucocorticoid hormones such as prednisone, progesterone and testosterone, are involved in the planarian regeneration regulation process in a specific way [15]. The effect of two sex steroid hormones, estradiol and testosterone, on the regeneration rate and mitotic activity of neoblasts was studied in the most detail. Differences in the nature of their effects were found; estradiol had a stimulating effect, and testosterone, on the contrary, an inhibitory effect on planarian recovery. At the same time, both hormones markedly increased the mitotic activity in both anterior and posterior regenerants [16].

The data on the morphogenetic activity of glucocorticoids are fragmentary and need more detailed study.

To study the functional activity of a certain hormone, the antagonists and/or agonists thereof are often used to see whether they are able to compete with the studied hormones for binding to their receptors. Some of these synthetic analogues are capable of acting both as antagonists and agonists of the studied hormones depending on the concentration. In this case, their ways of interacting with hormone receptors change, and this leads to the development of different physiological effects. A well-known concentration dependent agonist and antagonist is methylprednisolone [17]. A comparative functional analysis of a hormone alongside its antagonists and agonists allows us to understand the effect of the studied steroids more precisely.

Steroid hormones typically act through nuclear receptors. The family of nuclear steroid hormone receptors includes androgen-, estrogen-, and gluco-mineralocorticoid receptors. The manifestation of a specific hormonal effect is determined by the hormone-receptor interaction of a single hormone with its corresponding nuclear receptor, as well as the nature of the acceptor—the target organ cells [16]. The presence of receptors for one or another group of steroids in the studied species can be an indirect confirmation of its inclusion in the various processes involved in the regulation mechanism, including regeneration.

The aim of our research was to study the morphogenetic effects of the steroid hormones: hydrocortisone and its synthetic analogue methylprednisolone, capable of acting as an agonist and antagonist of hydrocortisone, in regenerating *Girardia tigrina* planarians. To achieve this goal, we set the following tasks:

(1) To study the effect of hydrocortisone in the concentration range from 10^−3^ M to 10^−6^ M and methylprednisolone in the concentration range from 10^−4^ M to 10^−6^ M on the reparative regeneration process of the head and tail planarian blastemas (new unpigmented tissue formed at the regeneration site);
(2) To study the effect of exogenous steroid hormones hydrocortisone and methylprednisolone on the proliferation of planarian neoblast stem cells;
(3) To search for homologs of glucocorticoid receptor proteins in a closely related species *Schmidtea mediterranea*, with fully sequenced and annotated genome.

## 2. Materials and Methods

### 2.1. The Object

The laboratory culture of the asexual planarian species *Girardia tigrina* (*Turbellaria*, *Tricladida*) was used as a model organism for the experiments. Planarians were kept in a darkened room at a temperature of 20–23 °C. Feeding was performed once a week by larvae of chironomids (Katrinex, Warsaw, Poland). In the experiments, planarians with a body length of 9–11 mm were used. Previously, they were kept without feeding for 7 days.

The effect of two steroid hormones of the glucocorticoid group on the reparative regeneration process was studied: hydrocortisone and a synthetic hormone of this group—methylprednisolone, a well-known agonist of glucocorticoid receptors and to a lesser extent of mineralocorticoids [18].

The hydrocortisone stock (Sigma, H0888, Saint Louis, MO, USA) in DMSO (Sigma, Saint Louis, MO, USA) at a concentration of 10^−1^ M or methylprednisolone (Supelco, PHR1717, Bellefonte, PA, USA) in DMSO in concentration of 10^−1^ M was added into a 50 mL glass with planarians in an amount sufficient to achieve the needed concentration.

The control and experimental group were kept in 50 mL of a solution of tap and distilled water (in a 2:1 ratio). In each glass, 30 individuals were kept for each experimental and control group. The test compound solutions were added to the experimental groups, to achieve the needed concentrations. The experiments were conducted in three independent series.

### 2.2. Morphometric Analysis

The regeneration results were evaluated using the method of lifetime computer morphometry [19]. The method is based on the presence of a clear boundary between the non-pigmented blastema—a section of regenerating tissue and the pigmented main part of the body. An OLYMPUS CX41 LF microscope equipped with an OLYMPUSU-CMAD3 digital camera was used for everyday planarian imaging. Images of the formed blastema area and the entire regenerating fragment were processed with the Plana 4.4 program (author’s development).

The decapitation and tail removal of planarians was performed as follows. The animals were placed upon a cooled (+6 °C) platform, stationed under a binocular microscope, (MBS-10, Moscow, Russia). At a low temperature the planarians stop moving, which facilitates the operation. The operation was performed with a scalpel under 20× magnification.

Four concentrations of hydrocortisone: 10^−3^ M, 10^−4^ M, 10^−5^ M and 10^−6^ M, and three concentrations of methylprednisolone: 10^−4^ M, 10^−5^ M and 10^−6^ M were used in the experiments. The experiment lasted for 6 days. Measurements were conducted every 24 h for 4 days in the period from the 3rd to the 6th day of the experiment. The recovery rate of the blastema formed as a result of either decapitation (anterior blastema) or tail area removal (posterior blastema) was estimated. As a quantitative criterion for the development of the blastema and the assessment of the dynamics of the recovery of the original proportions of the animal body, the regeneration coefficient (RC) calculated by the formula was used:

RC = s/S(1)

where: s—the blastema area; S—the animal’s body area.

### 2.3. Immunohistochemical Analysis

The method of immunohistochemical labeling of neoblasts was used to identify mitotic cells in the planarian body [20]. The planarians were treated for 5 min in 7% N-acetylcysteine. After that, the animals were fixed in 4% PBS-formaldehyde solution with 0.3% Triton X100 (Sigma, Saint Louis, MO, USA) for 20 min. Primary antibodies to phosphorylated histone H3 (Santa Cruz, Dallas, Texas, USA) at a 1/300 dilution and secondary antibodies with fluorescent dye CF488A (Biotium, Fremont, CA, USA) at a 1/500 dilution were used.

After washing the planarians of secondary antibodies, they were placed in a Vectashield immunofluorescence medium (Vector Labs, Newark, CA, USA) and visualized using an AxioScope A1 microscope (Carl Zeiss, München, Germany) equipped with an AxioCamMRc camera (Carl Zeiss, München, Germany). The calculation of the number of dividing cells and the planarian body area was performed using the ImageJ program. 

The mitotic index (MI) was used as a criterion of neoblasts’ mitotic activity. It was calculated by the formula:

MI = n/S,(2)

where: n—the number of cells; S—the animal’s body area, mm^2^.

**Figure 1:**
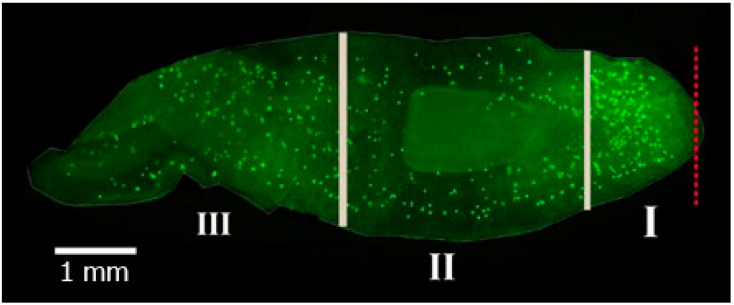
Zones of recovering planarian body. (**I**–**III**)—the number of the zone. The red dotted line indicates the amputation line.

Only the decapitated worms with regenerating head blastema (Figure 1) were used for a detailed study of the neoblast distribution in the planarian body and the nature of the hormones’ effect on their proliferative activity in different body areas. They were conditionally subdivided into three zones along the anteroposterior axis: I—the periwound zone, II—the zone near the pharynx, III—the tail zone. Mitotic activity of dividing cells in planarians in control and experimental groups was calculated in each zone (Figure 1).

### 2.4. The Search for Homologues of Receptor Proteins

As the *G. tigrina* genome has not been sequenced yet, we have performed a search for orthologous proteins of glucocorticoid receptors (hydrocortisone) in the genome of a closely relates species, namely *Schmidtea mediterrana*, using the SmedGD genomic database (https://planosphere.stowers.org/smedgd, accessed on 17 November 2022). We used the BLAST algorithm for this, which was also used previously to search for amino acid sequences based on similarity with the target protein receptors in humans (*Homo sapiens*), house mouse (*Mus musculus*), African clawed frog (*Xenopus laevis*), and fruit fly (*Drosophila melanogaster*). The search was performed both by the complete amino acid sequence and on the basis of ligand-binding domains.

The homologues found in the planarian genome were also compared in the GenBank database and the most similar sequences were selected. Domain analysis in the protein structure was determined using the NCBI Conserved domains database. Next, multiple alignment of amino acid sequences was performed with the ClustalW algorithm using an online service EMBL (European Molecular Biology Laboratory: //www.ebi.ac.uk/, accessed on 21 October 2022).

Phylogenetic comparisons of amino acid sequences were performed using an online service, EMBL (European Molecular Biology Laboratory: //www.ebi.ac.uk/, accessed on 21 October 2022).

### 2.5. Statistical Data Processing

Statistical analysis of the obtained results was performed using the SigmaPlot 11 program. The parametric Student’s *t*-test was used at a significance level of 0.95%. The results are presented as averages ± standard deviation. 1290 individuals were used in the study.

## 3. Results

### 3.1. Dynamics of Changes in the Regeneration Coefficient of the Regenerating Head and Tail Blastema with Hydrocortisone

**Figure 2:**
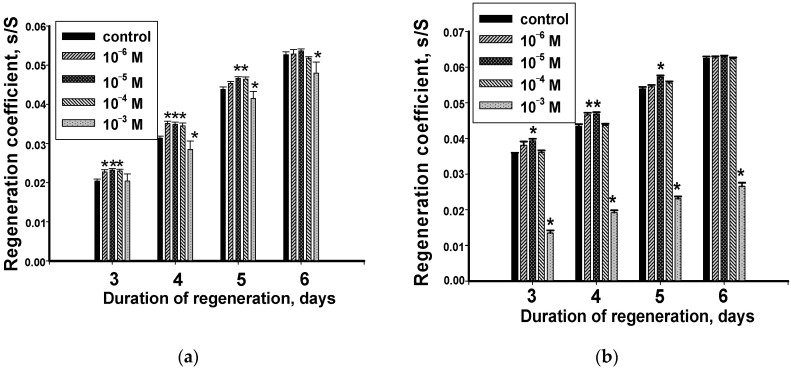
The hydrocortisone effect on the regeneration coefficient of the head (**a**) and tail (**b**) blastema (*n* = 30, 3 independent experiments). *—significant difference with the control *p* ≤ 0.05. Data are presented as mean ± sem.

In regenerating head blastema, the highest of the studied concentrations of hydrocortisone (10^−3^) involved in the regeneration regulation process started from 4 days after amputation (Figure 2a). During the entire period of research, it steadily inhibited blastema growth. The growth dynamics of the regenerating head blastema with the hormone did not change; as with the control, a gradual increase in the size of the regenerating head blastema was observed during regeneration.

The three lower concentrations of hydrocortisone (10^−6^, 10^−5^ and 10^−4^ M) had a pronounced stimulating effect on the course of the regeneration process in head blastema (Figure 2a). The duration of their influence on the rate of recovery was shorter than in the previous case and persisted only for five days during the experiment. It should be noted that while maintaining the positive nature of the effect of the lowest hormone concentration (10^−6^), the differences in the blastema size in experimental and control animals in this solution on the fifth day of the experiment were not significant (Figure 2a).

The worms with regenerating tail blastemas who retained cerebral ganglia after amputation were also particularly sensitive to the highest hydrocortisone concentration (10^−3^) (Figure 2b). The high content of the hormone had a pronounced inhibitory effect on recovering animals during the observation period. At the same time, with the increased duration of hormone action, which was involved in the regulation of growth processes on the third day of the experiment, the intensity of its effect significantly increased. It led to a decrease in the RC values relative to the control ones.

The worms with regenerating tail blastemas were less sensitive to low concentrations of the added hormone. Hydrocortisone at a concentration of 10^−4^ M had practically no effect on the regeneration processes of the tail blastema. The effect of the hormone at a concentration of 10^−6^ M had a significant stimulating effect only during the second day of the experiment. Only in a solution containing hydrocortisone at concentration of 10^−5^ M, was the stimulating effect of the hormone observed, and it persisted for five days of the experiment. On the sixth day, the values of RC in the control and experimental groups of planaria were represented by similar values (Figure 2 b).

### 3.2. Dynamics of Changes in the Mitotic Index with Hydrocortisone in Various Zones of Head Blastema Regenerants

**Figure 3:**
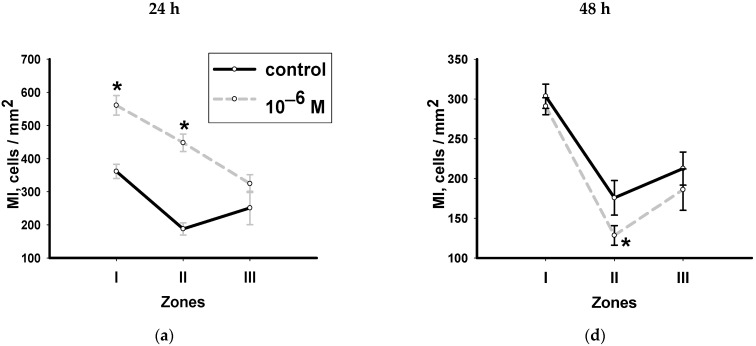

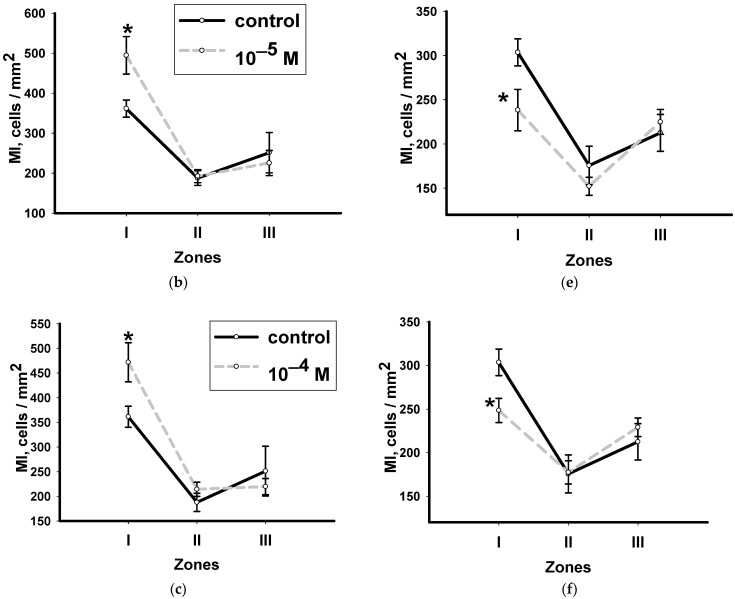
Effect of hydrocortisone at concentrations of 10^−6^ M (**a**,**d**), 10^−5^ M (**b**,**e**), 10^−4^ M (**c**,**f**) on the neoblast proliferation activity in various zones of worms with regenerating head blastema, 24 and 48 h after decapitation (*n* = 10, 3 independent experiments). *—significant difference with the control *p* ≤ 0.05. Data are presented as mean ± sem.

Hydrocortisone at all concentrations, stimulated the proliferative activity of neoblasts near the amputation location (zone I) 24 h after decapitation. The maximum values of MI were recorded with the lowest hydrocortisone concentration of (10^−6^ M), (Figure 3a). Higher concentrations of hydrocortisone (10^−5^ and 10^−4^ M) also stimulated the proliferative activity of neoblasts in this zone, but the intensity of their effect on this process was much lower than in the first case, (Figure 3b,c).

The tail zone farthest from the amputation site (zone III) turned out to be the least sensitive to the hormone. The studied concentrations did not significantly affect the proliferative activity of the neoblasts located here (Figure 3a–c).

A day after decapitation (48 h), the nature of the hormone’s effect on the proliferative activity of neoblasts changed. In the decapitation zone (zone I), the hormone at a concentration of 10^−6^ M practically did not affect the number of stem cells: the values of MI in animals were similar to those in the control group, (Figure 3d). The hydrocortisone effect at concentrations of 10^−5^ and 10^−4^ M changed to the opposite: both concentrations reduced the rate of mitosis. A particularly noticeable inhibitory effect of the hormone was registered under the influence of the hormone at a concentration of 10^−5^ M (Figure 3e).

The hormone at a concentration of 10^−6^ M had a significant inhibitory effect on the mitotic activity of neoblasts in the near-pharyngeal zone (zone II) (Figure 3d). An increase in the concentration of hydrocortisone to 10^−5^ M, led to a decrease in the degree of inhibitory effect of the hormone (Figure 3e). The addition of the highest concentration of hormones (10^−4^ M) to the medium did not affect the change in the number of dividing cells (Figure 3f).

### 3.3. Dynamics of Changes in the Regeneration Coefficient of Head and Tail Blastema with Methylprednisolone

**Figure 4:**
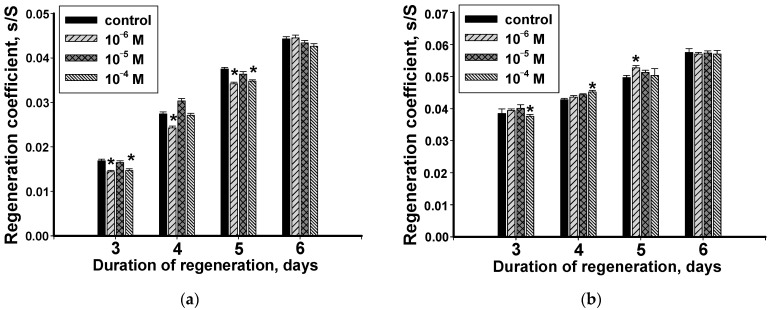
The effect of methylprednisolone on the regeneration coefficient of the regenerating head (**a**) and tail (**b**) blastema (*n* = 30, three independent experiments). *—a significant difference with the control *p* ≤ 0.05. Data are presented as mean ± sem.

In the first days after surgery (day 3), the lowest and highest concentrations (10^−6^ M and 10^−4^ M) inhibited the regeneration process of the head blastema (Figure 4a). The average of the hormone concentrations (10^−5^ M) did not affect the growth activity of the blastema. This type of hormone exposure persisted for five days of the experiment. It should be noted that the effect of the highest concentration of methylprednisolone (10^−4^ M) on the fourth day of the experiment was not statistically significant. By the sixth day, all the experimental concentrations of the hormone did not have a significant effect on the regeneration process course: the RC values in animals from the experimental groups were close to the control ones (Figure 4a).

The recovery of the regenerating tail blastema was low depending on methylprednisolone (Figure 4b). On the 3rd day after surgery only the highest concentration (10^−4^ M) changed the rate of blastema growth. Its effect was inhibitory, and the next day (day 4) changed to the opposite; the hormone stimulated the growth of the blastema. The lowest hormone concentration was involved in the regeneration regulation process only by the fifth experimental day. An increase in the regeneration rate was observed in experimental media. As a result, the RC indicators in planarians significantly exceeded the control values. At the final stage of the experiment (day 6), none of the concentrations affected the recovery rate of the tail blastema (Figure 4b).

### 3.4. Dynamics of Changes in the Mitotic Index with Methylprednisolone in Various Zones of Head Blastema Regenerants

**Figure 5:**
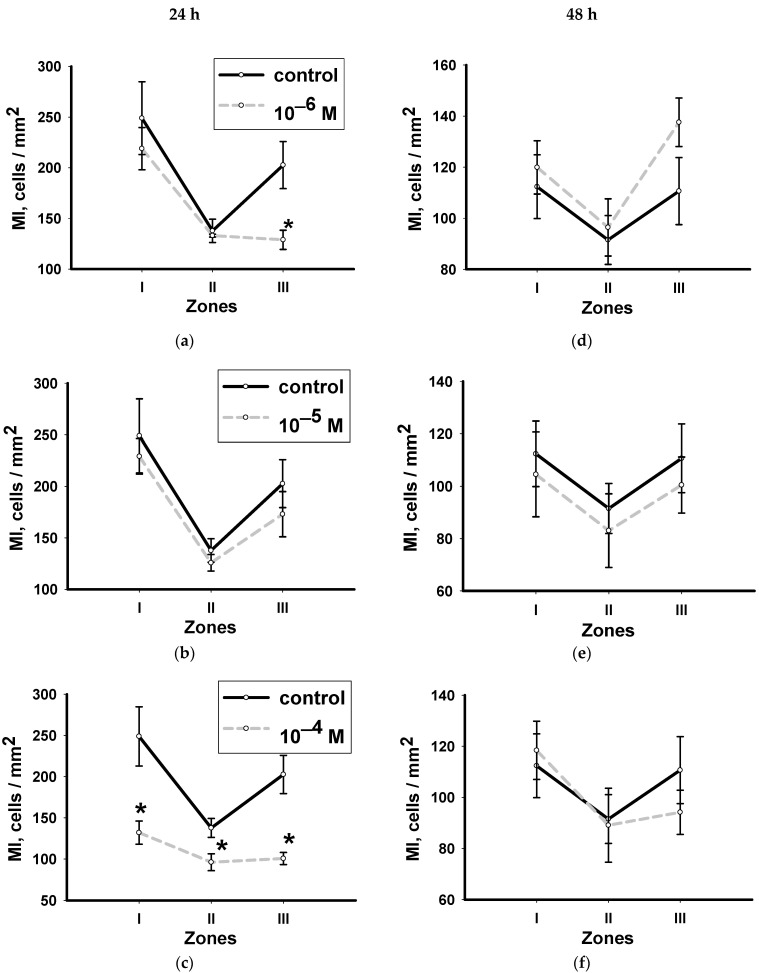
The effect of methylprednisolone at concentrations of 10^−6^ M (**a**,**d**), 10^−5^ M (**b**,**e**), 10^−4^ M (**c**,**f**) on the activity of neoblast proliferation in various zones of posterior regenerants 24 and 48 h after decapitation (*n* = 10, 3 independent experiments). *—a significant difference with the control *p* ≤ 0.05. Data are presented as mean ± sem.

The highest concentration of the hormone (10^−4^ M) had a pronounced inhibitory effect a day after amputation (24 h). The MI values in all the studied zones of the worm body in the experiment were significantly lower than in the control. Particularly low values of neoblast mitotic activity were recorded in zone I and zone III (Figure 5c).

Statistical analysis showed that the average concentration (10^−5^ M) was not practically involved in cell division regulation in any of the studied sites on the regenerating fragment. The differences in the MI in experimental and control animals were not consistent (Figure 5b).

A further decrease in the concentration of methylprednisolone in the media had practically no effect on the neoblast proliferative activity in the periwound (zone I) and near-pharyngeal zones (zone II). In the tail zone (zone III), the hormone significantly inhibited this process (Figure 5a).

None of the methylprednisolone concentrations had a statistically significant effect on the mitogenic activity of neoblasts 48 h after amputation (Figure 5d–f).

### 3.5. The Search for Homologs of Steroid Hormone Receptor Proteins

An orthologous protein of the glucocorticoid receptor was detected using a BLAST algorithm search in the SmedGD genomic database of *Schmidtea mediterranea*. It also has a high degree of homology to another nuclear receptor—nuclear liver factor 4.

**Figure 6:**
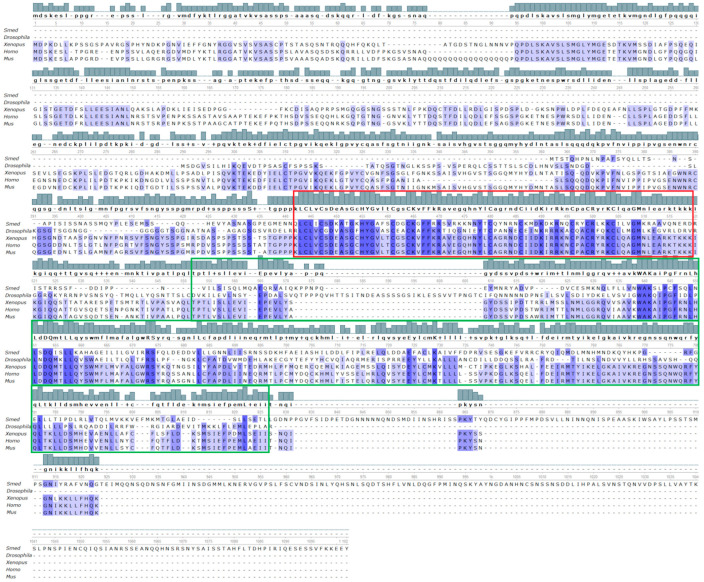
Multiple alignment of the glucocorticoid receptor of *S. mediterranea* (Smed) and the receptor proteins of *D. melanogaster* (Drosophila), African clawed frog (*Xenopus laevis*) (Xenopus), human (*Homo sapiens*) (Homo) and mouse (*Mus musculus*) (Mus). The DNA binding domain and ligand binding domain are highlighted in red and green boxes, respectively. Identical amino acids are shown in navy blue. The alignment is made on the consensus sequence of the human protein. The degree of homology of the receptor orthologue with a human sequence reaches 57.8%. Alignment was performed using the ClustalW algorithm.

The glucocorticoid receptor discovered in the *S. mediterranea* genome consists of 665 amino acids (Figure 6). Analysis of the nuclear receptor domain structure in the NCBI database showed the presence of domains typical for this receptor type—a zinc-binding domain which is located from 65 to 132 amino acids, domains responsible for binding to DNA (located from 66 to 141 amino acids), as well as a ligand-binding domain (160–377 amino acids), (Figure 6). Moreover, the homology of these domains has a high degree of statistical significance (2.80 × 10^−40^, 4.34 × 10^−45^ and 2.63 × 10^−78^, respectively).

The multiple alignment of the amino acid sequence of the glucocorticoid receptor planarian orthologue demonstrated that this protein has a 57.8% homology with the consensus sequence of the human glucocorticoid receptor protein (Figure 6).

**Figure 7:**
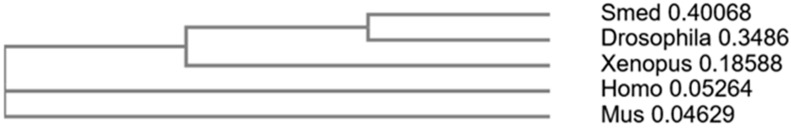
Phylogenetic analysis of the amino acid sequence of the glucocorticoid receptor of *S. mediterranea* (Smed) and *D. melanogaster* (Drosophila) receptor proteins, of the African clawed frog (*Xenopus laevis*) (Xenopus), human (*Homo sapiens*) (Homo) and mouse (*Mus musculus*) (Mus). The analysis was performed using the MrBayes program.

Phylogenetic analysis has shown that the glucocorticoid receptors of flatworms, as well as *Drosophila*, are quite far from the group of vertebrate glucocorticoid receptor orthologs but they descend from a common ancestor. The results are consistent with the data on the taxa origin, since the cladogram traces the origin of the glucocorticoid receptor from a common ancestor in amphibians, as well as the later isolation of the avian and mammalian receptor (Figure 7).

## 4. Discussion

Our studies have shown that hydrocortisone and methylprednisolone are able to influence the course of the morphogenetic process in planarians. The nature of planarian recovery depended on the qualitative specificity of the hormone, the concentration of each of them and the regenerating fragment (head or tail blastema).

The identified inhibitory effect of hydrocortisone is confirmed by the data of classical endocrinology, where the hormone is described mainly as an anti-growth one [21]. Currently, it has been shown that hydrocortisone possesses an inhibitory effect on the differentiation and viability of nerve stem cells in vertebrates, leading to their necrosis and apoptosis [22]. Hydrocortisone in high doses can inhibit fetal growth during vertebrate embryogenesis [23]. 

It was shown previously, that the nature of hydrocortisone action may vary depending on its concentration. In vitro experiments demonstrated that lower hormone concentrations can stimulate growth and anabolic processes in the connective tissue structures of vertebrates, and higher ones can suppress the same processes [24,25]. This may explain the stimulating effect of the low concentration of hydrocortisone that we observed in our study. Hydrocortisone has been independently shown to enhance the proliferation and regeneration of epithelial cells in vertebrates in a concentration-dependent matter [26,27].

The effect of methylprednisolone on regeneration activity was studied before only in a range of low concentrations (from 10^−4^ to 10^−6^ M). Unlike hydrocortisone, they had different effects on the degree and nature of the responses of the planarian regenerating blastema. In medical practice, methylprednisolone and its derivatives are known as synthetic steroids widely used as anti-inflammatory drugs or immunosuppressants [28]. The dependence of the direction and intensity of the effect of this drug on the concentration is described in detail in the medical literature [29,30].

While studying growth of the regenerating head blastema in methylprednisolone solutions, it was found that two of the concentrations (10^−4^ and 10^−6^ M) had an inhibitory effect, as opposed to hydrocortisone. The concentration of 10^−5^ M turned out to be inactive.

For the regenerating head blastema less sensitive to the introduction of the hormone, there is not enough data for general conclusions about the nature of its effect on the regeneration rate. In cases where a comparison of similar concentrations of two hormones was possible, it was found that one of the concentrations of methylprednisolone (10^−5^ M) was inactive, in contrast to the stimulating effect of hydrocortisone. The nature of the effect of the highest concentration of methylprednisolone (10^−4^ M) at the beginning of the experiment changed from negative to positive over time, while hydrocortisone was not involved in the regulation of planarian recovery during this period.

To explain these data, additional studies are needed. It can only be assumed that the differences revealed in the effects of the two hormones are associated with the ability of methylprednisolone to act both as an agonist and an antagonist of hydrocortisone as it does in vertebrates. In both cases, the mechanism of its action depends on the ability to bind to glucocorticoid receptors. The interaction of the antagonist with the receptor prevents the release of the receptor from complexes with proteins, thus preventing the translocation of the ligand–receptor complex into the nucleus. However, some complexes manage to reach the target DNA, but then the transcriptional activity is significantly reduced [17]. As an agonist, the hormone, having affinity for the receptor, modifies the receptor protein itself, thereby enhancing its activity. The biological effectiveness of agonists, i.e., their effect on cell functions, depends on the degree of receptor activation influence on the signal transmission in the cell. The nature of the interaction of methylprednisolone with receptors may change over time [18]. It is possible that the indicated differences in the effects of similar hydrocortisone and methylprednisolone concentrations are explained by the ability of methylprednisolone to interact with hydrocortisone receptors and the dependence of its effects on the type of this interaction; as an agonist or an antagonist.

We found an orthologous protein of the glucocorticoid receptor in the SmedGD genomic database of the *Schmidtea mediterranea* planaria. It turned out that it also has a high homology to another nuclear receptor—hepatocyte nuclear factor 4 (HNF4). Based on the results, it can be proposed that flatworms have nuclear receptors for glucocorticoid hormones. All of them have rather conservative domains that characterize the performed functions. The specific effects of each hormone that we have identified may be associated with the formation of specific hormone–receptor complexes. They include a cascade of biochemical reactions characteristic only for such a given complex.

The differences we found in the response to the added hormones in the anterior and posterior regenerants may be related to differences in their morphogenetic patterns. Their formation in all planarian tissues normally and during regeneration processes is ensured by the work of “positional control genes” encoding such signaling pathways as: Wnt, FGF, BMP and Hedgehog (Hh). The Wnt signaling pathway controls the correct restoration of the anterior and posterior poles during planarian regeneration and is activated immediately after injury. At the same time, the main proteins of the Wnt pathway are expressed at the posterior pole, and the Wnt antagonist protein notum is expressed only at the anterior pole [31].

Steroid hormones can be included in the expression regulation of different genes at the level of nuclear receptors, thereby changing the work of the corresponding signaling pathways in the regenerating planarian blastema. This may be related to the identified features of the studied steroids’ effects on the recovery of the anterior and posterior structures of planaria. Another reason for the different sensitivity to hormones of recovering areas may be the features of the disto–proximal distribution of receptors in the planarian body [32,33].

The morphogenetic effect of glucocorticoid group steroids indicated by us in *Girardia tigrina* is confirmed by data obtained from other planarian species. The treatment of the *Dugesia lugubris* planarian by prednisone—the dehydrated analogue of hydrocortisone had a stimulating effect on their growth which lasted until the complete finish of regeneration. The effect was more pronounced in the anterior regenerants than in the posterior ones [15].

The data on the effect of glucocorticoids on a range of physiological processes in other invertebrate taxa could be another confirmation of the possibility of this group of hormones to be involved in the regulation of the growth process in planarians. For example, in *Daphnia magna* crustaceans, it had a stimulating effect on somatic growth and reproductive activity [9]. In bivalves, hydrocortisone regulated the rate of gametogenesis processes (Ottaviani et al., 1998). When studying the effect of hydrocortisone on the growth and development of flies from the family *Sarcophagidae*, a stimulating effect of this hormone was found [34]. 

The addition of two sex steroids—estradiol and testosterone—into the media with *Girardia tigrina* planaria also influenced the course of regeneration in the head and tail blastema. The anterior regenerants were more sensitive to these hormones, they retained the head ganglion after amputation. The effect of these hormones on the regeneration process rate differed: estradiol had a stimulating effect, and testosterone, on the contrary, had an inhibitory effect on the recovery process [16].

Such sensitivity to steroid hormones can also be explained by the presence of a presumably two-level endocrine system in flatworms. It includes two ways of humoral regulation of various body functions: (1) with the help of neurosecretory compounds synthesized in different parts of the nervous system and (2) using different groups of steroid hormones [2,6,7,35]. The presence of glucocorticoids has been established in a number of parasitic flatworms by qualitative and quantitative methods [14,36,37]. There is evidence of the possibility of endogenous steroid synthesis in representatives of this group and its physiological significance (synthesis enzymes and significance for parasite worms [38]).

In free-living *Plathelminthes*, the neurosecretory system is most well studied, in parasitic species—the steroid profile. The steroid hormones, namely, hydrocortisone and testosterone, were detected and quantified in the *Fasciola hepatica* parasitic flatworm in concentrations of 0.001223 ug and 0.01193 ug per g of body weight, respectively [14].

In another three parasitic flatworm species, *Clonorchis sinensis*, *Opisthorchis viverrini* and the blood fluke *Schistosoma haematobium*, the steroid compounds have been found by means of HPLC and mass spectrometry: namely the oxysterols and catechol estrogens [35].

The flatworms (*Cestoda*) possess their own systems of steroidogenesis. So, the *Taenia crassiceps* worms are able to transform androstendione into testosterone in in vitro experiments, which was proven by recrystallization studies. In vitro experiments have also shown that steroidogenesis blocking compounds stop the steroid synthesis in flatworms, which confirms the presence of enzymatic pathways responsible for sex steroid synthesis in these animals [36].

The cultivation of *Taenia crassiceps* in the presence of dehydroepiandrosterone, tritium-labeled androstenedione, androstenediol and 17b oestradiol clearly demonstrate the presence and activity of the enzymes of the steroid biosynthesis pathway in this species [37].

The study of androgen’s role in the growth of *T. crassiceps* cysticerci has shown that addition of androgen antagonists into the culture lead to a significant reduction in protein biosynthesis. This fact allowed the conclusion that the ability to synthesize endogenic testosterone from its precursors is necessary for growth and development of the species [39]. Along with the ability of the cestodes to synthesize the sex steroids, the ability to synthesize corticosteroids has been revealed in the same species. The incubation of *T. crassiceps* flatworms in the presence of 3H progesterone has shown its transformation into deoxycorticosterone, a steroid, which possesses mostly mineralocorticoid properties in vertebrates with a slight glucocorticoid activity. In *Taenia solium,* an ability to synthesize glucocorticosteroids has been revealed by radioimmunological analysis [37,38].

Exogenous steroids can disrupt hormonal homeostasis in a planarian body, thereby starting a cascade of reactions associated with specific hormonal effects. In the case of injury, planarians trigger a wound reaction consisting of two waves. The first response occurs quickly—between 1 and 6 h after injury. It includes increased mitotic division of neoblasts throughout the planarian body and increased death of damaged cells near the wound. This reaction occurs independently of the injury type. Injuries associated with significant tissue loss cause a second wave of reactions which are called the missing tissue response. It occurs 40 to 48 h after the injury. Signs of the missing tissue response include: a second and sustained peak of mitosis in the periwound area and increased apoptosis throughout the body [40].

The ability of the hormones to influence regeneration processes is confirmed by the data we have obtained on their ability to influence the activity of neoblast proliferation. During the first mitotic peak, hydrocortisone significantly increased the rate of mitosis in the periwound zone. Methylprednisolone, on the contrary, mainly suppressed the proliferation of neoblasts and did so in the tail zone where normally their number is already small. The second peak is characterized by a complete absence of mitotic activity of methylprednisolone and a change in the nature of the hydrocortisone effect. Two hormone concentrations (10^−5^ and 10^−4^) suppressed the division of neoblasts in the postblastemic region.

Immunohistochemical labeling of neoblasts was performed using antibodies to phospho-histone H3. This histone is a marker of the transition from the G2 phase to the mitosis phase in the cell cycle. Obviously, hydrocortisone is able to both shorten and increase the duration of this transition. Methylprednisolone acted only as an inhibitor in this process.

## 5. Conclusions

1. Hydrocortisone and its synthetic analogue methylprednisolone are able to influence the course of the morphogenetic process in the *Girardia tigrina* planarian. There is a dependence of the nature and exposure degree of hormones on their qualitative specificity, the concentration and the regenerating fragment; the head or tail area. The anterior regenerants have less sensitivity to the studied hormones.
2. The nature of the hydrocortisone effect on the regeneration process differs from its synthetic analogue methylprednisolone which can act as an antagonist and an agonist of hydrocortisone. A high concentration of hydrocortisone suppresses the recovery of the amputated area, and low ones stimulate it. The nature of the methylprednisolone effect in the range of concentrations similar to the hydrocortisone ones, was more dependent on the specific concentration of the hormone and was mainly inhibitory.
3. Both hormones change the mitotic activity of neoblasts during the first peak of proliferation. Their effects are the opposite: hydrocortisone stimulates the process in the periwound zone, and methylprednisolone, on the contrary, inhibits it mainly in the zone furthest from the wound. The second wave of mitotic activity does not depend on methylprednisolone, and hydrocortisone inhibits the proliferation of neoblasts.
4. An orthologous protein of the glucocorticoid receptor was found in the SmedGD genomic database of the *Schmidtea mediterranea* planaria. Based on the obtained results, it can be proposed that flatworms have nuclear receptors for glucocorticoid steroid hormones. All of them have conservative domains that characterize the performed functions and probably play a role in many biological processes in planarians.
5. It can be assumed as a working hypothesis that exogenous hormones from the glucocorticoid group affect the endogenous mechanisms of endocrine regeneration, including through hormone–receptor interaction. The revealed features of the effects of hydrocortisone and methylprednisolone may be associated with the formation of specific hormone–receptor complexes, as well as with the dependence of the methylprednisolone effects on the type of interaction; as an agonist or an antagonist.

## Data Availability

Not applicable.
